# Bicarbonate induces high-level resistance to the human antimicrobial peptide LL-37 in *Staphylococcus aureus* small colony variants

**DOI:** 10.1093/jac/dkx433

**Published:** 2017-12-04

**Authors:** Ping Zhang, John A Wright, Anna Tymon, Sean P Nair

**Affiliations:** 1 Department of Microbial Diseases, UCL Eastman Dental Institute, 256 Gray’s Inn Road, London WC1X 8LD, UK; 2 Immunology Catalyst, GSK, Stevenage, Hertfordshire, UK

## Abstract

**Objectives:**

*Staphylococcus aureus* small colony variants (SCVs) cause persistent infections and are resistant to cationic antibiotics. Antimicrobial peptides (AMPs) have been suggested as promising alternatives for treating antibiotic-resistant bacteria. We investigated the capacity of the human cationic AMP LL-37 to kill SCVs in the presence of physiological concentrations of bicarbonate, which are reported to alter bacterial membrane permeability and change resistance of bacteria to AMPs.

**Methods:**

MBCs of LL-37 for *S. aureus* SCVs with mutations in different genes in the presence and absence of bicarbonate were determined.

**Results:**

In the absence of bicarbonate, SCVs of *S. aureus* strains LS-1 and 8325-4 had the same level of resistance to LL-37 as the parental strain (8 mg/L). In the presence of bicarbonate, *hemB*, *menD* and *aroD* SCVs of LS-1 had high-level resistance to LL-37 (≥128 mg/L) compared with the parental strain (16 mg/L). However, only the *aroD* SCV of strain 8324-5 showed high-level resistance. 8325-4 harbours mutations in two genes, *tcaR* and *rsbU*, which are involved in antimicrobial sensing and the stress response, respectively. When *rsbU* was repaired in 8325-4 it displayed high-level resistance to LL-37 in the presence of bicarbonate. This phenotype was lost when *tcaR* was also repaired, demonstrating that RsbU and TcaR are involved in LL-37 resistance in the presence of bicarbonate

**Conclusions:**

*S. aureus* SCVs would be resistant to high concentrations of LL-37 in niches where there are physiological concentrations of bicarbonate and therefore this AMP may not be effective in combating SCVs.

## Introduction

Small colony variants (SCVs) are slow-growing subpopulations of bacteria. *Staphylococcus aureus* SCVs are associated with persistent and recurrent infections such as osteomyelitis, implant infections, airway infections in cystic fibrosis patients^1^ and patients receiving long-term antibiotic treatment.^2–4^*S. aureus* SCVs isolated from patients are often found to be auxotrophic for metabolites, such as haemin and menadione.^5^ These two auxotrophs arise due to spontaneous mutations in genes encoding proteins in the haem and menadione biosynthesis pathways, with the most commonly studied mutations being in *hemB* or *menD*.^6^ Haem and menadione are required for the biosynthesis of some components of the electron transport chain (ETC). Therefore the ETC is interrupted in haemin or menadione auxotrophic SCVs.^7^ A functional ETC generates an electrochemical gradient (ΔΨ) across the cytoplasmic membrane, which is required for the uptake of positively charged molecules including positively charged antimicrobials such as aminoglycosides.^8^ The interrupted ETC in SCVs results in reduced uptake of aminoglycosides and hence increased resistance to aminoglycosides.

Given that most natural AMPs are cationic, there is the possibility that *S. aureus* SCVs will be more resistant to these molecules than the strains from which they arise. In fact, *S. aureus* SCVs have been reported to have increased resistance to a number of cationic AMPs including protamine,^9^ thrombin-induced platelet microbicidal protein^10^ and bovine lactoferricin B.^11^ To complicate matters AMPs could select for AMP-resistant SCVs. Pranting and Andersson^12^ reported that the cationic AMP protamine selects for *Salmonella enterica* serovar Typhimurium SCVs, which were found to be more resistant to a range of other AMPs, including colistin, lactoferricin and the human defensin HNP-1.

It has recently been reported that *S. aureus* SCVs have decreased susceptibility to a few skin-derived antimicrobial peptides.^13^ In this study the authors also examined the activity of LL-37 against SCVs and found there were strain-dependent differences in the susceptibility. Three strains that had undefined genetic mutations resulting in the SCV phenotype had between 2- and 4-fold reduced susceptibility to LL-37, while a defined SCV mutant with a disruption in *hemB* was no less susceptible to LL-37.^13^ Because the mutations in three of the SCVs were not defined, the variance in LL-37 susceptibility found could have been due to strain differences or due to different gene mutations giving rise to the SCV in the different strains. Aside from the skin, LL-37 is expressed by many cell types, in different tissues and in body fluids.^14^ The antimicrobial activity of LL-37 in various sites in the human body may vary since it has been shown that bacterial susceptibility to antimicrobial peptides is dependent on the ionic environment of the host. In particular bicarbonate has been suggested to be a crucial ionic factor affecting bacterial susceptibility to LL-37.^15^

In the study described here we examined the antimicrobial activity of the human cathelicidin LL-37 against *S. aureus* SCVs and determined if physiological levels of bicarbonate affected this activity.

## Materials and methods

### Bacterial strains and culture conditions

The bacterial strains used in this study are summarized in Table S1 (available as Supplementary data at *JAC* Online). *Escherichia coli* was grown under aerobic conditions in LB broth at 37 °C. *S. aureus* strains were grown under aerobic conditions in tryptic soy broth (Sigma–Aldrich, T8907) at 37 °C with shaking at 200 rpm. Erythromycin was added to 5 mg/L and chloramphenicol was added to 10 mg/L where required for propagation of bacteria. An SH1000 *rsbUVWsigB* mutant was constructed by phage transduction of the *rsbUVWsigB::ermB* mutation from *S. aureus* LS-1 *rsbUVWsigB::ermB* using ø85 as described by Nair *et al.*^16^ A markerless mutant of *aroD* in strain 8325-4 was constructed as described for strain LS-1 by Zhang *et al.*^6^

### MBC assays

The susceptibility of *S. aureus* WT and mutants to LL-37 in the presence or absence of bicarbonate was determined by measuring MBCs. The MBC assay buffer was a modified version of that described by Dorschner *et al.*^15^ and consisted of 1 mM NaH_2_PO_4_ with or without 50 mM NaHCO_3_ at pH 7.4. MBC assays were performed according to a method described previously.^3^ Briefly, ∼1 × 10^4^ cells from overnight bacterial cultures were inoculated into 100 μL of assay buffer containing LL-37 at 0, 1, 2, 4, 8, 16, 32, 64 or 128 mg/L. Samples were incubated at 37 °C for 2 h and then serial dilutions were plated onto tryptone soya agar to determine the number of viable cells. The numbers of the viable cells in each well were compared with the control that had no LL-37 and the MBC was defined as the lowest concentration resulting in ≥99.9% kill.

## Results

### Susceptibility of SCVs to LL-37 in the presence and absence of bicarbonate

To replicate mutations that give rise to *S. aureus* SCVs in clinical isolates we constructed and/or used pre-existing *S. aureus* SCVs with disruptions or deletions in three genes, *aroD*, *hemB* or *menD* . The *hemB* gene encodes 5-aminolevluniate dehydratase, an enzyme that is required for haem biosynthesis and hence cytochrome function. The *menD* gene encodes 2-succinyl-5-enolpyruvyl-6-hydroxy-3-cyclohexene-1-carboxylate synthase, an enzyme involved in menaquinone biosynthesis. The gene *aroD* encodes 3-dehydroquinate dehydratase, which is part of the shikimate pathway and is responsible for the synthesis of chorismate, a branching point for the biosynthesis of menaquinone, aromatic amino acids and several other metabolites. Therefore, all three mutants have a defective ETC.

The MBCs of LL-37 for the *S. aureus* WT strains LS-1 and 8325-4, and their isogenic SCVs, in the presence or absence of bicarbonate are summarized in Table 1. All of the SCVs had the same susceptibility to LL-37 as their respective WT strain in the absence of bicarbonate. In the presence of bicarbonate, the two WT strains behaved differently, with the susceptibility of LS-1 to LL-37 decreasing 2-fold, while that of 8325-4 remained the same. All of the SCVs derived from LS-1 demonstrated a >8-fold increase in resistance to LL-37 (MBC > 128 mg/L) compared with the WT strain (MBC = 16 mg/L). Interestingly, of the 8325-4 derived strains, only the SCV defective in *aroD*, but not those with *hemB* or *menD* mutations, showed a high level of bicarbonate-induced resistance to LL-37 (MBC = 128 mg/L). Complementing a functional *aroD* gene into both LS-1 Δ*aroD* and 8325-4 Δ*aroD* negated the bicarbonate-dependent resistance to LL-37. The reason for the difference in response to bicarbonate by the SCVs of the two different WT strains must have been due to genetic differences in the lineages. It is established that strain 8325-4 is defective in RsbU and TcaR,^17^ and we have established that LS-1 is not.^16^ RsbU is a positive regulator of the alternate sigma factor, σ^B^, which is involved in response to stress.^18^ TcaR is a regulator of the teicoplanin-associated operon *tcaRAB*, which has been implicated in resistance to the antibiotics teicoplanin and methicillin,^19^ but it is also a positive regulator of the global regulatory element SarS and the virulence factor protein A.^17^ We therefore focused on these genetic differences between the two lineages to see if they accounted for the difference in bicarbonate-inducible LL-37 resistance in the SCVs of these two strain backgrounds.

**Table 1.** MBC (mg/L) of LL-37 for different *S. aureus* SCVs in sodium phosphate buffer or in sodium phosphate buffer supplemented with bicarbonate

Strain	1 mM NaH_2_PO_4_	1 mM NaH_2_PO_4_ +NaHCO_3_
LS-1	8	16 (16–32)
LS-1 Δ*hemB*	8	>128
LS-1 Δ*meD*	8	>128
LS-1 Δ*aroD*	8	>128
LS-1 Δ*aroD* pSK236::*aroD*	8	16 (8–16)
8325-4	8 (4–8)	8 (8–16)
8325-4 Δ*hemB*	8	8 (8–16)
8325-4 Δ*menD*	8	8 (8–16)
8325-4 Δ*aroD*	8	128 (64–128)
8325-4 *ΔaroD* pSK236::*aroD*	8 (4–8)	16 (16–32)

The values in parentheses represent the range of MBC.

### Effect of deleting the σ^B^ operon on bicarbonate-induced resistance to LL-37

The susceptibility of SH1000 *rsbUVWsigB*, which is essentially 8325-4 with a deletion of the entire alternate sigma factor operon, and LS-1 *rsbUVWsigB* in the presence and absence of bicarbonate was determined and compared with the WT strains, as summarized in Table 2. Deletion of the entire *sigB* operon had no effect on the susceptibility of the 8325-4 lineage to LL-37 in the presence or absence of bicarbonate. Upon deletion of the *sigB* operon in LS-1 there was a 2-fold increase in the susceptibility to LL-37 in the absence of bicarbonate; however, this mutant still responded to bicarbonate with a 2-fold decrease in susceptibility to LL-37. These results suggested that the *sigB* operon may not be important in bicarbonate-induced high-level resistance to LL-37.

**Table 2.** Comparison of the MBC (mg/L) of LL-37 for *S. aureus* strains, with or without an intact *rsbU* gene, in sodium phosphate buffer or in sodium phosphate buffer supplemented with bicarbonate

Strain	1 mM NaH_2_PO_4_	1 mM NaH_2_PO_4_ + NaHCO_3_
LS-1	8	16 (16–32)
LS-1 Δ*rsbUVWsigB*	4 (4–8)	8 (4–16)
8325-4 (*rsbU*^−^)	8 (4–8)	8
SH1000 (*rsbU*^+^)	8	>128
SH1000 Δ*rsbUVWsigB*	8	8

The values in parentheses represent the range of MBC.

### Effect of repairing rsbU in strain 8325-4 on bicarbonate-induced resistance to LL-37

Unexpectedly we found that SH1000 (RsbU-repaired 8325-4) had high-level resistance to LL-37 in the presence of bicarbonate (Table 2; MBC > 128 mg/L). This was unexpected because LS-1, which has an intact *rsbU*, does not display bicarbonate-inducible high-level resistance to LL-37. The finding that repairing the *rsbU* defect in 8325-4 resulted in bicarbonate-inducible high-level resistance to LL-37 demonstrates that the *rsbUVWsigB* operon does play a role in this process. Since LS-1 has an intact *rsbUVWsigB* operon and does not display this phenotype, it suggested that the TcaR defect in SH1000 may also play a role in bicarbonate-inducible high-level resistance to LL-37.

### Mutants defective in tcaR display bicarbonate-dependent high-level resistance to LL-37, which can be abolished by either repairing tcaR or deleting sigB

**Figure 1.**
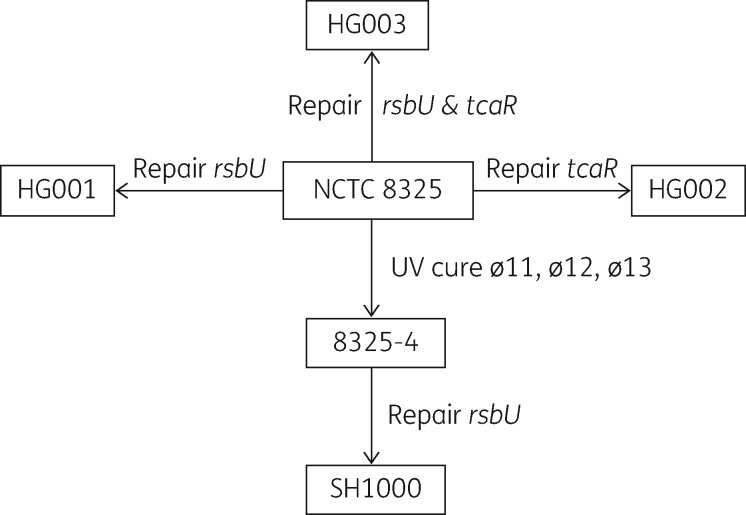
Relationship between the NCTC 8325 lineage strains.

To determine if TcaR accounts for the difference in bicarbonate-induced high-level resistance to LL-37 between strains LS-1 and SH1000, a set of strains derived from the NCTC 8325 lineage were utilized (Figure 1).^17^ Strain NCTC 8325 is known to be defective in both *rsbU* and *tcaR*, and contains three prophages ø11, ø12 and ø13. Strain 8325-4 used in the initial experiments described here is a derivative of NCTC 8325 cured of the prophages. Strains HG001, HG002 and HG003 were modified directly from strain NCTC 8325 by repairing *rsbU*, *tcaR* or both genes, respectively.^17^ The MBC of LL-37 for each of these isogenic strains was determined and the results are shown in Table 3. All of the strains had a similar level of resistance to LL-37 in the absence of bicarbonate. As found in previous experiments in this study, the two *rsbU^−^tcaR^−^* strains, 8325-4 and SH1000 Δ*rsbUVWsigB*, did not have high levels of resistance to LL-37 in the presence of bicarbonate. In all strains where *rsbU* was repaired, but in which *tcaR* was defective, i.e. strains SH1000 and HG001 (*rsbU*^+^*tcaR*^−^), the presence of bicarbonate-induced high-level resistance to LL-37 (MBC ≥ 128 mg/L). This suggests that RsbU, through regulation of σ^B^, positively regulates the bicarbonate-inducible high-level resistance to LL-37 phenotype in *tcaR*-defective strains. When *tcaR* was repaired in a strain competent for RsbU, i.e. strain HG003 (*rsbU*^+^*tcaR*^+^), the bicarbonate-inducible high-level resistance to LL-37 phenotype was lost. This suggests that TcaRAB and SigB operons have opposite effects in the regulation of bicarbonate-inducible high-level resistance to LL-37. Furthermore, the *rsbU*^−^*tcaR*^+^ strain HG002 showed a similar level of resistance to LL-37 in the presence of bicarbonate as the *rsbU*^+^*tcaR*^+^ strain HG003, demonstrating that when TcaR is functioning, deleting or repairing *rsbU* does not affect the high-level resistance to LL-37 phenotype in the presence of bicarbonate. From these data it is apparent that bicarbonate does not induce the high-level resistance to LL-37 phenotype in strains with a *tcaR*^+^ genotype and only strains with the genotype *rsbU*^+^*tcaR*^−^ exhibit bicarbonate-dependent high-level resistance to LL-37.

**Table 3.** Comparison of the MBC (mg/L) of LL-37 for *S. aureus* strains of the NCTC 8325 lineage, with or without defects in the *rsbU* and/or *tcaR* genes, in sodium phosphate buffer or in sodium phosphate buffer supplemented with bicarbonate

Strain	Genotype	1 mM NaH_2_PO_4_	1 mM NaH_2_PO_4_ + NaHCO_3_
8325-4	*rsbU*^−^, *tcaR*^−^	8 (4–8)	8
SH1000	*rsbU*^+^, *tcaR*^−^	8	>128
SH1000 Δ*rsbUVWsigB*	*rsbU*^−^, *tcaR*^−^	8	8
HG001	*rsbU*^+^, *tcaR*^−^, ø11, ø12, ø13	8	128
HG002	*rsbU*^−^, *tcaR*^+^, ø11, ø12, ø13	8	16 (16–32)
HG003	*rsbU*^+^, *tcaR*^+^, ø11, ø12, ø13	8	16 (16–32)

The values in parentheses represent the range of MBC.

## Discussion

*S. aureus* SCVs are a subpopulation of cells that are slow growing and able to survive and persist in the host.^1^ One of the important features of SCVs is an increased resistance to aminoglycosides^20^ conferred by a disrupted ETC.^21^ To investigate whether a defective ETC also increases SCV resistance to cationic AMPs, the activity of LL-37 against *S. aureus* haemin and menadione auxotrophs was determined. SCVs that had mutations in *hemB*, *menD* or *aroD* were found to be as susceptible to LL-37 as their parental strains, demonstrating that the disrupted ETC in these SCVs, which would result in reduced membrane potential, does not confer resistance to LL-37. These findings to a small extent contrast with those of Glaser *et al.*^13^ who found that three out of four SCVs that they examined had slightly increased resistance to LL-37. However, in their study, the nature of the genetic change conferring an SCV phenotype on the three SCVs that had increased resistance to LL-37 was not defined, nor was it determined if these strains contained additional mutations. The SCV strain that did not have an increased resistance to LL-37 was a defined mutant that was identical to the 8325-4 *hemB* mutant used in this study. Therefore possible explanations for the findings of Glaser *et al.*^13^ could include: (i) SCV phenotypes that result from different gene mutations have different levels of resistance to LL-37; (ii) SCVs in different strain backgrounds have different levels of resistance to LL-37; and (iii) the SCV strains that had increased resistance to LL-37 had additional mutations, unrelated to the SCV phenotype, which were responsible for the increased resistance to LL-37. Since we examined SCVs that arose from different genetic mutations that are commonly isolated in clinical settings the first explanation above seems unlikely. The second explanation also seems unlikely since we used very different strain backgrounds and did not find increased resistance to LL-37. Hence the third explanation that there were additional mutations in the genome of the SCVs, unrelated to the SCV phenotype, that conferred increased resistance to LL-37 seems more likely; however, none of the other explanations can be ruled out completely.

In the presence of physiological concentrations of bicarbonate the *hemB*, *menD* and *aroD* SCVs of *S. aureus* LS-1 all demonstrated high levels of resistance to LL-37 compared with the WT strain. While an *aroD* SCV of strain 8325-4 had high-level resistance to LL-37 in the presence of bicarbonate, neither the *hemB* SCV nor the *menD* SCV of this strain had increased resistance. Our findings that the WT *S. aureus* strains either had no change in resistance or increased resistance to LL-37 in the presence of bicarbonate are at odds with the data published by Dorschner *et al.*^15^ who found that bicarbonate increased the susceptibility of *S. aureus* to LL-37. We hypothesized that the strain-dependent differences in the resistance of the LS-1 and 8325-4 SCVs to LL-37 in the presence of bicarbonate were probably due to known defects in two genes, *rsbU* and *tcaR*, in the NCTC 8325 strain lineage. Indeed, when *rsbU* was repaired in strains of the NCTC 8325 lineage they demonstrated high-level resistance to LL-37 in the presence of bicarbonate. Repairing *tcaR* or inactivating *rsbU* abolished high-level resistance to LL-37 in the presence of bicarbonate. RsbU is a positive regulator of the alternate sigma factor σ^B^, which is important for *S. aureus* responses to environmental stress, survival and virulence.^22^^,^^23^ Expression of σ^B^ is increased in *S. aureus* SCVs and has been reported to be the major regulator of virulence in cells of this phenotype.^24^ Overexpression of σ^B^ leads to increased cell wall thickness^25^ and it is tempting to speculate that this may in part account for the high-level resistance to LL-37 seen in SCVs in the presence of bicarbonate. TcaR, the teicoplanin-associated locus regulator, is a MarR family protein initially identified as the regulator of a teicoplanin-resistance-associated locus, which consists of *tcaR*, *tcaA* and *tcaB* (*tcaRAB*).^19^ TcaR is now recognized to be a multifunctional regulator, regulating genes involved in polysaccharide intercellular adhesin production^26^ and *sarS*, a member of the global regulatory network.^27^ Deletion of the *tcaRAB* locus leads to increased resistance to glycopeptide antibiotics and some clinical isolates of glycopeptide-intermediate-resistant *S. aureus* (GISA) harbour mutations in the *tcaRAB* locus, which accounts for this resistance.^28^ Whilst it is clear that defects in the *tcaRAB* operon can give rise to clinical GISA, the exact mechanisms behind this are not known. GISA have thickened cell walls with altered compositions and it has been suggested that these thickened cell walls sequester glycopeptides before they can reach their membrane located target lipid II.^29^^,^^30^ The GISA phenotype has also been linked to low-level resistance to cationic thrombin-induced platelet microbicidal proteins.^31^ However, in the case of LL-37 the thickened cell wall on its own cannot account for the high-level resistance of *S. aureus*, since physiological levels of bicarbonate are required for this phenotype. Mirroring our findings on bicarbonate-induced high-level resistance to LL-37 in *S. aureus*, σ^B^ plays an important role in increased resistance to teicoplanin and vancomycin in GISA that have a defect in the *tcaRAB* locus.^28^ Taken together these data suggest that some clinical GISA will have an increased resistance to LL-37 *in vivo* and may therefore have a survival advantage. Exactly how σ^B^ regulates glycopeptide resistance is not known, but it does so through a secondary regulator encoded by the *yabJ*-*spoVG* operon.^32^

In summary, the data generated in this study highlight that the physiological ionic component bicarbonate needs to be factored into studies on the resistance of bacteria to antimicrobial peptides. Our data also infer that some GISA may have high-level resistance to LL-37 *in vivo*.

## Supplementary Material

Supplementary Data

## References

[1] von Eiff C, Peters G, Becker K. The small colony variant (SCV) concept—the role of staphylococcal SCVs in persistent infections. Injury 2006; 37 Suppl 2: S26–33. 16651068 10.1016/j.injury.2006.04.006

[2] Proctor RA, van Langevelde P, Kristjansson M Persistent and relapsing infections associated with small-colony variants of *Staphylococcus aureus*. Clin Infect Dis 1995; 20: 95–102. 7727677 10.1093/clinids/20.1.95

[3] von Eiff C, Bettin D, Proctor RA Recovery of small colony variants of *Staphylococcus aureus* following gentamicin bead placement for osteomyelitis. Clin Infect Dis 1997; 25: 1250–1. 9402396 10.1086/516962

[4] von Eiff C, Becker K, Metze D Intracellular persistence of *Staphylococcus aureus* small-colony variants within keratinocytes: a cause for antibiotic treatment failure in a patient with darier’s disease. Clin Infect Dis 2001; 32: 1643–7. 11340539 10.1086/320519

[5] Kahl BC. Small colony variants (SCVs) of *Staphylococcus aureus*–a bacterial survival strategy. Infect Genet Evol 2014; 21: 515–22. 23722021 10.1016/j.meegid.2013.05.016

[6] Zhang P, Wright JA, Osman AA An *aroD* ochre mutation results in a *Staphylococcus aureus* small colony variant that can undergo phenotypic switching via two alternative mechanisms. Front Microbiol 2017; 8: 1001. 28620368 10.3389/fmicb.2017.01001 PMC5449664

[7] McNamara PJ, Proctor RA. *Staphylococcus aureus* small colony variants, electron transport and persistent infections. Int J Antimicrob Agents 2000; 14: 117–22. 10720801 10.1016/s0924-8579(99)00170-3

[8] Baumert N, von Eiff C, Schaaff F Physiology and antibiotic susceptibility of *Staphylococcus aureus* small colony variants. Microb Drug Resist 2002; 8: 253–60. 12523621 10.1089/10766290260469507

[9] Sadowska B, Bonar A, von Eiff C Characteristics of *Staphylococcus aureus*, isolated from airways of cystic fibrosis patients, and their small colony variants. FEMS Immunol Med Microbiol 2002; 32: 191–7. 11934563 10.1111/j.1574-695X.2002.tb00553.x

[10] Koo SP, Bayer AS, Sahl HG Staphylocidal action of thrombin-induced platelet microbicidal protein is not solely dependent on transmembrane potential. Infect Immun 1996; 64: 1070–4. 8641763 10.1128/iai.64.3.1070-1074.1996 PMC173884

[11] Samuelsen O, Haukland HH, Kahl BC *Staphylococcus aureus* small colony variants are resistant to the antimicrobial peptide lactoferricin B. J Antimicrob Chemother 2005; 56: 1126–9. 16287983 10.1093/jac/dki385

[12] Pranting M, Andersson DI. Mechanisms and physiological effects of protamine resistance in *Salmonella enterica* serovar Typhimurium LT2. J Antimicrob Chemother 2010; 65: 876–87. 20233778 10.1093/jac/dkq059

[13] Glaser R, Becker K, von Eiff C Decreased susceptibility of *Staphylococcus aureus* small-colony variants toward human antimicrobial peptides. J Invest Dermatol 2014; 134: 2347–50. 24717245 10.1038/jid.2014.176

[14] Durr UH, Sudheendra US, Ramamoorthy A. LL-37, the only human member of the cathelicidin family of antimicrobial peptides. Biochim Biophys Acta 2006; 1758: 1408–25. 16716248 10.1016/j.bbamem.2006.03.030

[15] Dorschner RA, Lopez GB, Peschel A The mammalian ionic environment dictates microbial susceptibility to antimicrobial defense peptides. FASEB J 2006; 20: 35–42. 16394265 10.1096/fj.05-4406com

[16] Nair SP, Bischoff M, Senn MM The σ^B^ regulon influences internalization of *Staphylococcus aureus* by osteoblasts. Infect Immun 2003; 71: 4167–70. 12819110 10.1128/IAI.71.7.4167-4170.2003 PMC161998

[17] Herbert S, Ziebandt AK, Ohlsen K Repair of global regulators in *Staphylococcus aureus* 8325 and comparative analysis with other clinical isolates. Infect Immun 2010; 78: 2877–89. 20212089 10.1128/IAI.00088-10 PMC2876537

[18] Horsburgh MJ, Aish JL, White IJ σ^B^ modulates virulence determinant expression and stress resistance: characterization of a functional *rsbU* strain derived from *Staphylococcus aureus* 8325-4. J Bacteriol 2002; 184: 5457–67. 12218034 10.1128/JB.184.19.5457-5467.2002 PMC135357

[19] Brandenberger M, Tschierske M, Giachino P Inactivation of a novel three-cistronic operon *tcaR*-*tcaA*-*tcaB* increases teicoplanin resistance in *Staphylococcus aureus*. Biochim Biophys Acta 2000; 1523: 135–9. 11042376 10.1016/s0304-4165(00)00133-1

[20] Massey RC, Buckling A, Peacock SJ. Phenotypic switching of antibiotic resistance circumvents permanent costs in *Staphylococcus aureus*. Curr Biol 2001; 11: 1810–4. 11719226 10.1016/s0960-9822(01)00507-3

[21] Proctor RA, von Eiff C, Kahl BC Small colony variants: a pathogenic form of bacteria that facilitates persistent and recurrent infections. Nat Rev Microbiol 2006; 4: 295–305. 16541137 10.1038/nrmicro1384

[22] Pane-Farre J, Jonas B, Forstner K The *σ*^B^ regulon in *Staphylococcus aureus* and its regulation. Int J Med Microbiol 2006; 296: 237–58. 16644280 10.1016/j.ijmm.2005.11.011

[23] Nielsen JS, Christiansen MH, Bonde M Searching for small σ^B^-regulated genes in *Staphylococcus aureus*. Arch Microbiol 2011; 193: 23–34. 20978742 10.1007/s00203-010-0641-1

[24] Mitchell G, Fugère A, Pépin Gaudreau K SigB is a dominant regulator of virulence in *Staphylococcus aureus* small-colony variants. PLoS One 2013; 8: e65018. 23705029 10.1371/journal.pone.0065018 PMC3660380

[25] Morikawa K, Maruyama A, Inose Y Overexpression of sigma factor, ς^B^, urges *Staphylococcus aureus* to thicken the cell wall and to resist β-lactams. Biochem Biophys Res Commun 2001; 288: 385–9. 11606054 10.1006/bbrc.2001.5774

[26] Jefferson KK, Pier DB, Goldmann DA The teicoplanin-associated locus regulator (TcaR) and the intercellular adhesin locus regulator (IcaR) are transcriptional inhibitors of the *ica* locus in *Staphylococcus aureus*. J Bacteriol 2004; 186: 2449–56. 15060048 10.1128/JB.186.8.2449-2456.2004 PMC412131

[27] McCallum N, Bischoff M, Maki H TcaR, a putative MarR-like regulator of *sarS* expression. J Bacteriol 2004; 186: 2966–72. 15126456 10.1128/JB.186.10.2966-2972.2004 PMC400606

[28] Maki H, McCallum N, Bischoff M *tcaA* inactivation increases glycopeptide resistance in *Staphylococcus aureus*. Antimicrob Agents Chemother 2004; 48: 1953–9. 15155184 10.1128/AAC.48.6.1953-1959.2004 PMC415614

[29] Giuliani A, Pirri G, Nicoletto SF. Antimicrobial peptides: an overview of a promising class of therapeutics. Cent Eur J Biol 2007; 2: 1–33.

[30] Afacan NJ, Yeung AT, Pena OM Therapeutic potential of host defense peptides in antibiotic-resistant infections. Curr Pharm Des 2012; 18: 807–19. 22236127 10.2174/138161212799277617

[31] Sakoulas G, Eliopoulos GM, Fowler VG Jr Reduced susceptibility of *Staphylococcus aureus* to vancomycin and platelet microbicidal protein correlates with defective autolysis and loss of accessory gene regulator (*agr*) function. Antimicrob Agents Chemother 2005; 49: 2687–92. 15980337 10.1128/AAC.49.7.2687-2692.2005 PMC1168700

[32] Schulthess B, Meier S, Homerova D Functional characterization of the σ^B^-dependent *yabJ*-*spoVG* operon in *Staphylococcus aureus*: role in methicillin and glycopeptide resistance. Antimicrob Agents Chemother 2009; 53: 1832–9. 19223635 10.1128/AAC.01255-08 PMC2681525

