# Supplementary material for: Bicarbonate induces high-level resistance to the human antimicrobial peptide LL-37 in Staphylococcus aureus small colony variants
Source: J Antimicrob Chemother. 2017 Dec 4;73(3):615–9. doi: 10.1093/jac/dkx433 (PMC5890704; doi:10.1093/jac/dkx433)
Supplement: Supplementary Data [file supplementary_data_dkx433.doc]

**Supplementary data**

**Table S1.**

| Strains | Relevant characteristics | Reference |
| --- | --- | --- |
| *S. aureus RN4220* | Restriction deficient strain derived from *S. aureus* 8325-4 | 1 |
| *S. aureus* LS-1 | *S. aureus* septic arthritis isolate from mice | 2 |
| S. aureus LS-1 ΔhemB | *S. aureus* LS-1 with marker less deletion of *hemB* | 3 |
| *S. aureus* LS-1 Δ*menD* | *S. aureus* LS-1 with an insertion of *ermB* in *menD* | 4 |
| *S. aureus* LS-1 Δ*aroD* | *S. aureus* LS-1 with marker less deletion of  *aroD* nucleotide 448-717 | 4 |
| LS-1 *ΔaroD* pSK236 :: *aroD* | *S. aureus* LS-1 with marker less deletion of  *aroD* nucleotide 448-717 complemented with aroD | 4 |
| *S. aureus* LS-1 Δ*rsbUVWsigB* | *S. aureus* LS-1 with *rsbUVWsigB* replaced by *ermB* | 5 |
| *S. aureus* 8325-4 | Wild-type standard laboratory strain | 6 |
| *S. aureus* 8325-4 Δ*hemB* | *S. aureus* LS-1 with an insertion of *ermB* in *hemB* | 7 |
| *S. aureus* 8325-4 Δ*menD* | *S. aureus* LS-1 with an insertion of *ermB* in *menD* | 8 |
| *S. aureus* 8325-4 Δ*aroD* | *S. aureus* LS-1 with marker less deletion of  *aroD* nucleotide 448-717 | This study |
| *S. aureus* SH1000 | *S. aureus* 8325-4 with *rsbU* repaired | 9 |
| *S. aureus* SH1000 Δ *rsbUVWsigB* | *S. aureus* SH1000 with *rsbUVWsigB* replaced by *ermB* | This study |
| *S. aureus* HG001 | Essentially the same as *S. aureus* 8325-4 with *rsbU* repaired, but contains prophage *Ф11*, *Ф12* and*Ф13* | 10 |
| *S. aureus* HG002 | Essentially the same as *S. aureus* 8325-4 with tcaR repaired, but also contains prophage*Ф11*, *Ф12* and*Ф13* | 10 |
| *S. aureus* HG003 | Essentially the same as *S. aureus* 8325-4 with *rsbU* and tcaR repaired, but also contains prophage*Ф11*, *Ф12* and*Ф13* | 10 |

1. Kreiswirth BN, Lofdahl S, Betley MJ et al. The toxic shock syndrome exotoxin structural gene is not detectably transmitted by a prophage. *Nature* 1983; **305**: 709-12.

2. Bremell T, Lange S, Svensson L et al. Outbreak of spontaneous staphylococcal arthritis and osteitis in mice. *Arthritis Rheum* 1990; **33**: 1739-44.

3. Wright JA, Nair SP. The lipoprotein components of the Isd and Hts transport systems are dispensable for acquisition of heme by Staphylococcus aureus. *FEMS Microbiol Lett* 2012; **329**: 177-85.

4. Zhang P, Wright J, Osman A et al. An *aroD* Ochre Mutation Results in a *Staphylococcus aureus* Small Colony Variant that can Undergo Phenotypic Switching via Two Alternative Mechanisms. *Frontiers in Microbiology* 2017 in press.

5. Nair SP, Bischoff M, Senn MM et al. The sigma B regulon influences internalization of Staphylococcus aureus by osteoblasts. *Infect Immun* 2003; **71**: 4167-70.

6. Novick R. Properties of a cryptic high-frequency transducing phage in Staphylococcus aureus. *Virology*. United States, 1967; 155-66.

7. von Eiff C, Heilmann C, Proctor RA et al. A site-directed Staphylococcus aureus hemB mutant is a small-colony variant which persists intracellularly. *J Bacteriol* 1997; **179**: 4706-12.

8. Bates DM, von Eiff C, McNamara PJ et al. Staphylococcus aureus menD and hemB mutants are as infective as the parent strains, but the menadione biosynthetic mutant persists within the kidney. *J Infect Dis* 2003; **187**: 1654-61.

9. Horsburgh MJ, Aish JL, White IJ et al. sigmaB modulates virulence determinant expression and stress resistance: characterization of a functional rsbU strain derived from Staphylococcus aureus 8325-4. *J Bacteriol* 2002; **184**: 5457-67.

10. Herbert S, Ziebandt AK, Ohlsen K et al. Repair of global regulators in Staphylococcus aureus 8325 and comparative analysis with other clinical isolates. *Infect Immun* 2010; **78**: 2877-89.
